# ezTree: an automated pipeline for identifying phylogenetic marker genes and inferring evolutionary relationships among uncultivated prokaryotic draft genomes

**DOI:** 10.1186/s12864-017-4327-9

**Published:** 2018-01-19

**Authors:** Yu-Wei Wu

**Affiliations:** Graduate Institute of Biomedical Informatics, College of Medical Science and Technology, Taipei Medical University, No. 250, Wuxing St., Xinyi District, 110 Taipei, Taiwan

**Keywords:** Marker gene, Phylogenetic tree, Uncultivated species

## Abstract

**Background:**

Inferring phylogenetic trees for newly recovered genomes from metagenomic samples is very useful in determining the identities of uncultivated microorganisms. Even though 16S ribosomal RNA small subunit genes have been established as “gold standard” markers for inferring phylogenetic trees, they usually cannot be assembled very well in metagenomes due to shared regions among 16S genes. Using single-copy marker genes to build genome trees has become increasingly popular for uncultivated species. Predefined marker gene sets were discovered and have been applied in various genomic studies; however these gene sets might not be adequate for novel, uncultivated, draft, or incomplete genomes. The automatic identification of marker gene sets among a set of genomes with different assembly qualities has thus become a very important task for inferring reliable phylogenetic relationships for microbial populations.

**Results:**

A computational pipeline, ezTree, was developed to automatically identify single-copy marker genes for a group of genomes and build phylogenetic trees from the marker genes. Testing ezTree on a group of proteobacteria species revealed that ezTree was highly effective in pinpointing marker genes and constructing reliable trees for different groups of bacterial genomes. Applying ezTree to genomes that were recently recovered from metagenomes also showed that ezTree can help elucidate taxonomic relationships among newly recovered genomes and existing ones.

**Conclusions:**

The development of ezTree can help scientists build reliable phylogenetic trees for uncultivated species retrieved from environmental samples. The uncovered single-copy marker genes may also provide crucial hints for understanding shared features of a group of microbes. The ezTree pipeline is freely available at https://github.com/yuwwu/ezTree under a GNU GPLv3 license.

**Electronic supplementary material:**

The online version of this article (10.1186/s12864-017-4327-9) contains supplementary material, which is available to authorized users.

## Background

Metagenomics and single-cell genomics have been established as promising methods for mining and investigating novel organisms from a wide variety of environments. The term “microbial dark matter” was proposed to describe uncultivated organisms that can only be sequenced and studied from microbial communities [1], and a new view of the tree of life was proposed to plug more than 1000 newly recovered uncultivated genomes into existing phylogenetic trees [2]. Increasing numbers of studies have focused on analyzing novel genomes extracted from a huge variety of microbial communities [3–12], thus expanding and pushing our knowledge toward understanding these organisms and the roles they play in the environments.

One of the most popular techniques for investigating microbial communities is metagenomics, which seeks to directly obtain genomic sequences from the environments. Computational binning techniques [13–22] were developed to extract individual organisms directly from metagenomes. To understand the microbial diversity of the recovered genomes and place them in the tree of life, phylogenetic marker genes have been used to build trees for the newly identified species. 16S ribosomal RNA small-subunit genes, one of the most widely adopted phylogenetic markers, have been established as “gold standard” for probing the taxonomy of newly recovered organisms and constructing phylogenetic trees [23, 24]. However, due to shared regions of 16S rRNA genes, it is still a very challenging task for de Bruijn graph-based metagenomic assemblers, such as Meta-IDBA [25], SPAdes [26], Ray Meta [27], and MEGAHIT [28], to assemble intact 16S rRNA genes from metagenomes [29]. As a result, genomes recovered from metagenomes usually lack 16S genes (or consist of only very short gene fragments), making it impossible or very difficult to build phylogenetic trees using 16S sequences.

Whole-genome information was proposed for refining phylogenetic relationships between or among individual species [30–33]. Concatenated protein trees (trees based on combined protein data alignments) were proposed to compensate for 16S gene-based trees and are potentially more robust and informative [34]. In order to build concatenated protein trees, one needs to identify phylogenetic marker genes, defined as genes that appear once and only once in every organism considered in the study [35]. Genes satisfying this criterion have been used as markers for reliably reconstructing phylogenetic relationships for prokaryotic species, as demonstrated in previous studies [36, 37]. Various attempts have been made to discover such marker gene sets. For example, Ciccarelli et al. identified 31 marker genes in 191 bacterial species and built a highly resolved tree-of-life [36]. Different marker gene sets were also reported by other people [35, 37, 38]. The checkM software also discovered lineage-specific marker gene sets and used them to check the completeness and contamination ratios of prokaryotic genomes recovered from metagenomes [39].

Since individual genomes recovered from metagenomes are rarely complete, some of the genes from the predefined marker gene sets may be missing from the recovered genomes. Moreover, since constructing phylogenetic trees usually involves dozens or even hundreds of genomes, one may need to laboriously check the copy number of each gene in every genome in order to identify the marker gene set for building phylogenetic trees. Even though reliable gene prediction tools such as Prodigal [40] and FragGeneScan [41] were developed to alleviate efforts to predict genes from newly recovered prokaryotic genomes, tools to automatically identify marker genes in a group of genomes are still needed to infer taxonomic relationships for a set of genomes.

Herein, I introduce a computational pipeline for inferring marker genes and phylogenetic trees from a set of prokaryotic genomes. The pipeline takes a set of genomes, including newly recovered, fragmented, or incomplete ones, and is able to predict protein-coding genes from the input genomes, identify marker genes shared by all genomes, and produce concatenated protein alignments of marker genes along with a maximum-likelihood (ML) phylogenetic tree. Users with newly recovered genomes of any quality can very easily and effortlessly employ this pipeline to build a tree and infer the taxonomy of recovered species.

## Methods

**Fig. 1:**
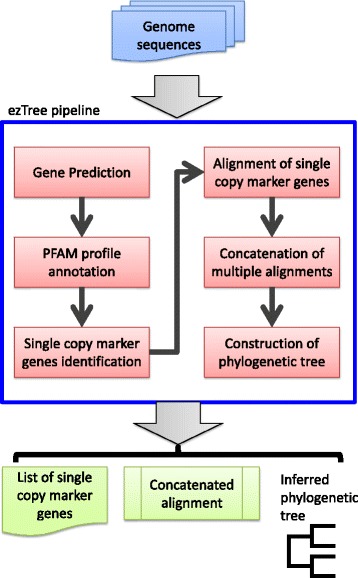
Workflow of ezTree to look for single-copy marker genes and use them to construct a phylogenetic tree for a set of input genomes

The pipeline was designed to take in a set of prokaryotic genomic sequences in fasta format. Genomic sequences can be complete, fragmented, or even incomplete. If users prefer, they may also input protein sequences instead of entire genomes. The workflow of the pipeline includes predicting protein-coding genes from the genomes, assigning functional profiles to the genes, identifying single-copy marker genes for the set of genomes, and aligning sequences to produce a phylogenetic tree, as depicted in Fig. 1. The implementation details of the pipeline are described below.

### Gene prediction and functional annotation

**Fig. 2:**
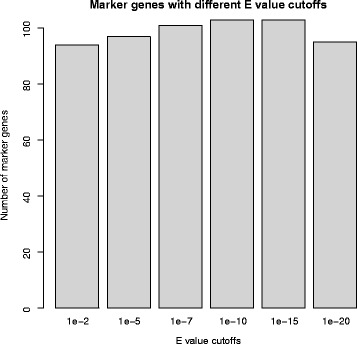
Number of single-copy marker genes extracted using different e-value cutoffs. The test dataset is the proteobacteria dataset listed in Additional file 1: Table S1

Gene prediction was performed using Prodigal [40] with parameter “-p meta” to accommodate novel or newly recovered fragmented genomes. The pipeline is able to check whether inputs are genomic sequences or proteins and skip the gene-prediction step for protein inputs. After extracting protein-coding genes from the genomes, the amino acid sequences were compared to PFAM hidden Markov models [42] using HMMER3 [43] with e-value cutoff set to 1e-10 (which was chosen to achieve a balance between sensitivity and specificity, as illustrated in Fig. 2, in which the greatest number of marker genes was identified at 1e-10 and 1e-15). Only the top hit for each gene was retained in order to preserve only the most likely mapping results and facilitate the search for single-copy marker genes.

### Single-copy marker gene identification

Gene annotations across all genomes were compared within and between genomes to look for marker genes. PFAM profiles that appeared more than once in each genome were discarded; the remaining profiles were further compared among all genomes. Only single-copy PFAM profiles that were found in all genomes were kept for further processing.

### Sequence alignment and phylogenetic tree construction

Once single-copy marker genes were identified for the set of input genomes, amino acid sequences of the genes were collected from all genomes and separately aligned using MUSCLE [44]. Alignments were then concatenated, one-by-one, to form a single alignment file. Gblocks [45] was further employed to remove highly variable or gapped positions in order to generate more-reliable trees. Finally FastTree [46] was used to generate an ML tree from the concatenated alignment with default options (JTT model, 1000 bootstraps).

### Pipeline output

Given a set of genomes, the pipeline was designed to identify 1) a list of marker genes; 2) a concatenated alignment file; and 3) the tree in the Newick format built by FastTree. The tree can be viewed using tools such as MEGA7 [47], TreeView [48], and FigTree [49]. If users wish, they can also take the alignment and use other tree-reconstruction software such as RAxML [50], Mr. Bayes [51], MEGA7 [47], PhyML [52], and IQ-TREE [53] to produce their own trees.

### Mapping PFAM profiles and cluster of orthologous groups (COG) categories

PFAM profiles and COG categories were mapped through the gene ontology (GO) website, which consists of COG-to-GO and PFAM-to-GO mapping results [54]. The mapping was done in two steps: 1) “cog2go” and “pfam2go” files were downloaded; and 2) COGs and PFAMs that could be mapped to the same GO terms were extracted. Definitions of COG categories were downloaded from the NCBI COG website [55].

### Availability

The pipeline along with a README and a tutorial PDF file is publicly available at https://github.com/yuwwu/ezTree under the GNU GPLv3 license. The set of Proteobacteria genomes used in the evaluation can also be downloaded from the github website.

## Results

**Fig. 3:**
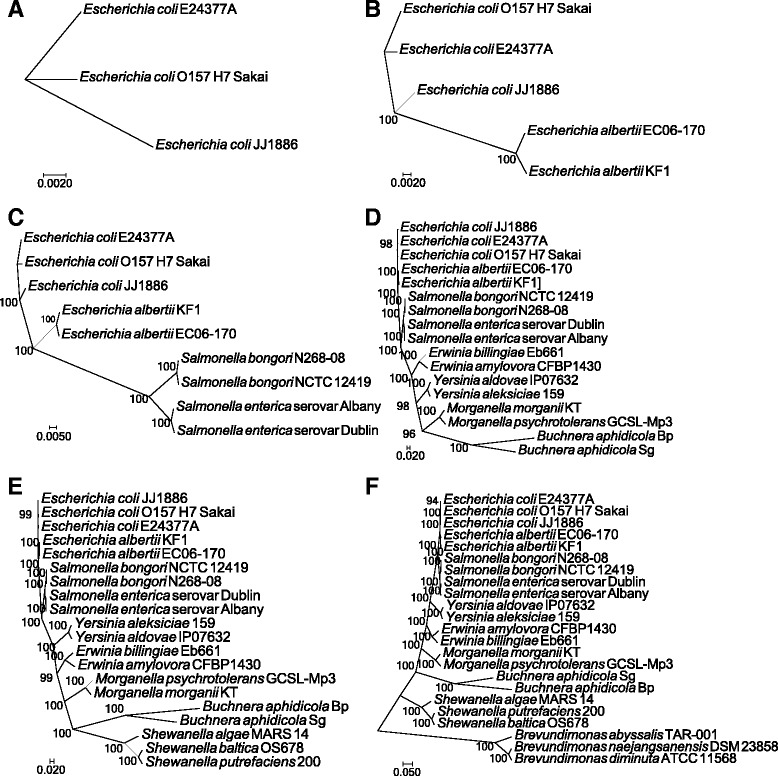
Reconstructed phylogenetic trees of a set of Proteobacteria genomes. Shared taxonomic ranks for each subset of genomes are (**a**) species; (**b**) genus; (**c**) family; (**d**) order; (**e**) class; and (**f**) phylum

The ezTree pipeline was first evaluated using 23 Proteobacteria genomes, among which six were draft genomes (i.e., genomes with more than one scaffold; detailed genome information is listed in Additional file 1: Table S1). ezTree successfully identified marker genes and built phylogenetic trees for genomes that shared the same species, genus, family, order, class, and phylum ranks, as shown in Fig. 3. Tree structures were consistent with known topologies, suggesting that ezTree was able to reconstruct phylogenetic relationships among the species. The high bootstrap support values for all branches of the trees indicate that trees built from the identified marker genes were very reliable.

**Fig. 4:**
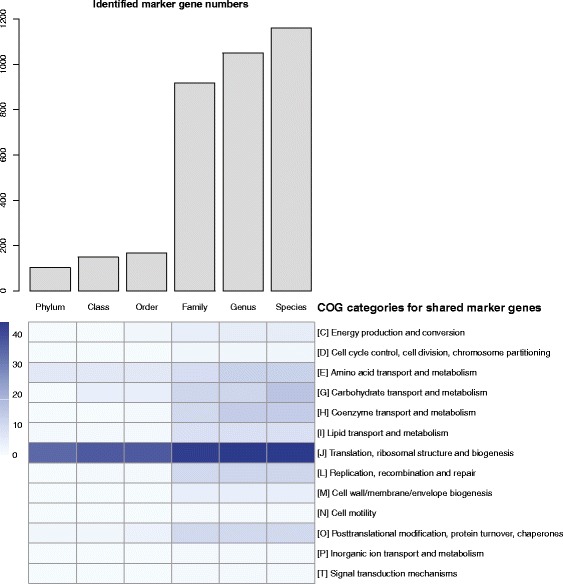
Numbers and COG annotations of the marker genes predicted from Proteobacteria genomes. Upper: The number of single-copy marker genes identified for Proteobacteria genomes shown in Fig. 3. Lower: Heatmap of predicted cluster of orthologous group (COG) categories of marker genes

Besides building trees, ezTree was also able to identify shared single-copy marker genes from the examined genomes. Numbers of identified marker genes are shown in the upper part of Fig. 4. As expected, genomes with the same species-, genus-, or family-level taxonomy shared many more marker genes (1161, 1051, and 917 marker genes, respectively, for species, genus, and family levels) than those with the same order-, class-, or phylum-level taxonomy (167, 149, and 103 marker genes, respectively, for order, class, and phylum levels). The COG categories of marker genes were identified by mapping PFAM profiles against COGs (see Implementation for details). One of the COG categories, “[J] Translation, ribosomal structure and biogenesis,” clearly stood out as the most abundant gene category for marker genes, as shown in the lower part of Fig. 4. This is consistent with other marker gene-related analyses, in which ribosomal proteins accounted for the majority of marker genes. For example, Huson et al. reported using 41 marker genes to guide gene-centric assembly of orthologous gene families, in which 30 of 41 (73%) marker genes were ribosomal proteins [56]. A new tree-of-life was also built on a set of 16 ribosomal protein sequences of organisms [2]. Note that only a fraction of genes can be mapped to COG categories due to the mapping between PFAM and COG; however, the consistency between this and past works cannot be overlooked.

**Fig. 5:**
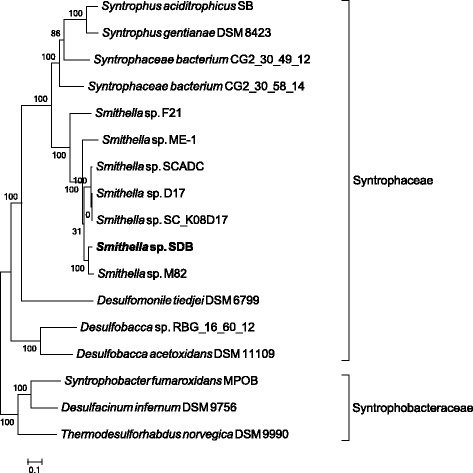
Reconstructed phylogenetic tree of a set of Syntrophobacterales genomes. The SDB genome described in Wawrik et al. [57] is highlighted in bold for clarity

The ezTree pipeline was also used to identify marker genes and phylogenetic relationships for several newly recovered genomes from metagenomes. In 2016, Wawrik et al. reported that the bacterial species *Smithella* sp. SDB coupled with hydrogenotrophic methanogens could degrade water-insoluble paraffins [57]. The draft genomes of *Smithella* sp. SDB, *Methanosaeta* sp. SDB, *Methanolinea* sp. SDB, and *Methanoculleus* sp. SDB were downloaded and applied ezTree to them along with other genomes downloaded from NCBI. For *Smithella* sp. SDB, ezTree successfully identified 31 marker genes (Additional file 1: Table S3) from a group of Syntrophobacterales, and the tree (Fig. 5; genome information is available in Additional file 1: Table S2) for the involved genomes was consistent with the 16S tree (Fig. 2 of Wawrik et al.’s paper [57]). Note that among the 17 Syntrophobacterales genomes, only four were complete genomes; the numbers of scaffolds of the draft genomes ranged from as low as 22 to as high as 1037. This clearly demonstrates the ability of ezTree to identify marker genes and build trees from draft genomes of any assembly quality.

**Fig. 6:**
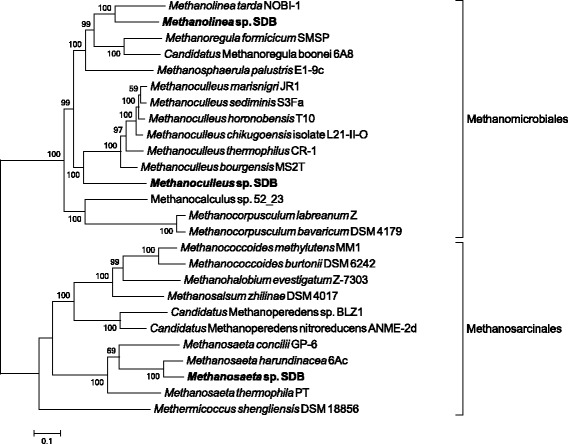
Reconstructed phylogenetic tree of a set of Methanomicrobia genomes. Three SDB genomes identified by Wawrik et al. [57] are highlighted in bold

ezTree also identified 75 single-copy marker genes for the three Methanomicrobia SDB genomes (genome information can be found in Additional file 1: Table S4; marker genes are listed in Additional file 1: Table S5). The tree built from concatenated proteins also clearly placed the three SDB genomes in their corresponding places, as shown in Fig. 6. Bootstrap values were very significant for most branches, lending support to the reliability of the constructed tree. It was interesting to observe that species of *Methanolinea* and *Methanosaeta* recovered by Wawrik et al. [57] were more closely related to known species, while the *Methanoculleus* sp. SDB was more distantly related to other *Methanoculleus*, hinting that the recovered *Methanoculleus* sp. SDB species may have the potential to become a new genus under the Methanomicrobiaceae family.

**Fig. 7:**
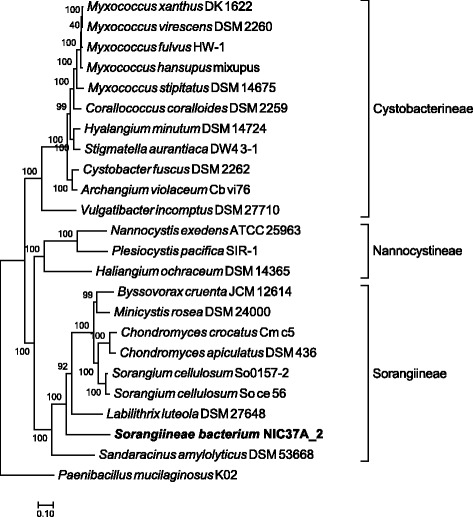
Reconstructed phylogenetic tree of a set of Myxococcales genomes. The recovered species, *Sorangiineae bacterium* NIC37A_2, is highlighted in bold for clarity

Last, ezTree was applied to a Myxococcales species recovered from enriched cellulolytic microbial consortia derived from green waste compost [22]. This recovered genome was the most abundant species in one of the two microbial communities and was found in 2014 to be distantly related to *Sorangium cellulosum*, as shown in Fig. 5 of Wu et al.’s MaxBin paper [22]. Applying ezTree to a set of Myxococcales genomes yielded 56 marker genes (Additional file 1: Table S7), and the resulting tree built from the marker genes (shown in Fig. 7) indicated that the recovered Myxococcales species was more closely related to *Labilithrix luteola* DSM 27648 and *Sandaracinus amylolyticus* DSM 53668, which were deposited in NCBI on August and May 2015, respectively. Detailed information of the involved genomes can be found in Additional file 1: Table S6. In other words, with more genomes deposited in NCBI, the Myxococcales species can now be pinpointed to the Sorangiineae suborder. The tree also hinted that the Myxococcales species probably does not belong to either *Labilithrix* or *Sandaracinus* genera, as the three species formed distinct branches on the tree. More genomes are still needed to fully uncover the identity of this uncultivated species.

## Discussion

A computational pipeline, ezTree, was developed to automatically infer single-copy marker genes and build reliable phylogenetic trees for a set of genomes. ezTree accepts both complete and draft genomes, including those with hundreds or even thousands of contigs or scaffolds, and is capable of automatically predicting and identifying phylogenetic marker genes. This functionality is very useful since increasing numbers of genomes are being recovered from metagenomes, and the first question we often ask is “what is it” when we are facing a new genome. ezTree thus provides an easy yet useful way to build trees and infer phylogenetic relationships with other species for newly recovered genomes.

One aspect worth noting is that ezTree needs no genome annotation information; it automatically infers annotations through the PFAM hidden Markov models. This feature relieves scientists of the burden of annotating genomes by themselves. In other words, after scientists obtain new prokaryotic genomes, they can put them—whether they are complete or are merely draft genomes—into the ezTree pipeline to infer the most likely taxonomic assignments of the novel species.

Identifying marker genes is a very important task in defining a taxonomic lineage; they can also be used to detect the completeness and contamination levels of genomes recovered from environmental samples. The ability of ezTree to identify marker genes provides scientists an easy route to investigate such information. For example, testing ezTree on collections of proteobacteria genomes and several newly identified genomes yielded highly reliable species trees, and different numbers of marker genes were also inferred by this process. These marker genes may be very important in defining distinct taxonomic ranks for a certain species, genus, family, order, class, or phylum.

Another issue related to ezTree is the selection of evolutionary models to build phylogenetic trees. Trees for the proteobacteria genomes and the Myxococcales genomes were built using different amino acid substitution models, including JTT, WAG, and LG, to test whether the selection of evolutionary models affects the tree topologies. Another option, Gamma20 model, which rescales the branch lengths and computes a Gamma20-based likelihood, was also included in the test. As shown in Additional file 1: Figure S1 and Figure S2, the trees using different amino acid substitution models are almost identical to each other, suggesting that issues related to model selection may be minor for common cases. The ezTree pipeline also provides an option to select models so that users may flexibly choose different evolutionary models or compare one model against the other.

To further validate marker gene sets, PFAM profiles of marker genes were mapped to COG categories. The greatest amount of mapped marker genes belonged to the category “[J] Translation, ribosomal structure, and biogenesis.” This result is consistent with other marker gene sets discovered by other groups, in which ribosomal proteins were indispensable in marker gene sets, and lent support to the robustness of the marker gene sets identified by ezTree.

With the help of ezTree, we can now infer more-accurate taxonomic assignments for newly recovered genomes. An example can be seen in the inferred tree of Myxococcales species recovered from adapted compost microbial communities. Without the availability of *Labilithrix luteola* DSM 27648 and *Sandaracinus amylolyticus* DSM 53668, we would only know that this species is distantly related to *Sorangium cellulosum* but have no idea about its actual taxonomy. Now we can safely put it in the Sorangiineae suborder since it closely grouped together with other genomes from this taxonomic lineage. Perhaps after more genomes are extracted either from pure cultures or from environmental samples and are deposited in NCBI, we can eventually designate a more-accurate taxonomy for this and other novel species.

## Conclusions

The ezTree pipeline can be used to extract marker genes and build concatenated-protein trees given a set of complete or draft genomes. Without prior knowledge except the genomic sequences, ezTree can infer single-copy marker genes for genomes and use the genes to build phylogenetic trees. Testing ezTree on multiple genome sets indicated that ezTree can be used to build highly reliable trees, providing crucial hints into defining the taxonomic lineages of the newly recovered prokaryotic genomes.

## Availability and requirements

Project name: ezTree v0.1.

Project Home Page: https://github.com/yuwwu/ezTree

Operating Systems: Linux.

Programming Language: Perl.

Other requirements: None.

License: GNU GPLv3.

Any Restrictions to Use By Non-Academics: None.

## Additional file

Additional file 1: Figure S1. The comparison of trees built for the set of Proteobacteria genomes provided by FastTree. **Figure S2.** The comparison of trees built for the set of Myxococcales genomes using different models provided by FastTree. **Table S1.** List of Proteobacteria genomes and their NCBI accession numbers used in the evaluation of ezTree. **Table S2.** List of Syntrophobacterales genomes and NCBI accession numbers used in inferring the tree for *Smithella* sp. SDB. **Table S3.** Single-copy marker genes identified for Syntrophobacterales genomes. **Table S4.** List of Methanomicrobia genomes and NCBI accession numbers used in inferring the tree for *Methanoculleus* sp. SDB, *Methanolinea* sp. SDB, and *Methanosaeta* sp. SDB. **Table S5.** Single-copy marker genes identified for Methanomicrobia genomes. **Table S6.** List of Myxococcales genomes and NCBI accession numbers used in inferring the tree for Sorangiineae bacterium NIC37A_2. **Table S7.** Single-copy marker genes identified for Myxococcales. (PDF 827 kb)

## Abbreviations

COG: Cluster of orthologous groups
GO: Gene ontology
NCBI: National Center for Biotechnology Information
PFAM: the protein family database
