# Supplementary material for: ezTree: an automated pipeline for identifying phylogenetic marker genes and inferring evolutionary relationships among uncultivated prokaryotic draft genomes
Source: BMC Genomics. 2018 Jan 19;19(Suppl 1):921. doi: 10.1186/s12864-017-4327-9 (PMC5780852; doi:10.1186/s12864-017-4327-9)
Supplement: Additional file 1: Figure S1. — The comparison of trees built for the set of Proteobacteria genomes provided by FastTree. Figure S2. The comparison of trees built for the set of Myxococcales genomes using different models provided by FastTree. Table S1. List of Proteobacteria genomes and their NCBI accession numbers used in the evaluation of ezTree. Table S2. List of Syntrophobacterales genomes and NCBI accession numbers used in inferring the tree for Smithella sp. SDB. Table S3. Single-copy marker genes identified for Syntrophobacterales genomes. Table S4. List of Methanomicrobia genomes and NCBI accession numbers used in inferring the tree for Methanoculleus sp. SDB, Methanolinea sp. SDB, and Methanosaeta sp. SDB. Table S5. Single-copy marker genes identified for Methanomicrobia genomes. Table S6. List of Myxococcales genomes and NCBI accession numbers used in inferring the tree for Sorangiineae bacterium NIC37A_2. Table S7. Single-copy marker genes identified for Myxococcales. (PDF 827 kb) [file 12864_2017_4327_MOESM1_ESM.pdf]

ezTree: an automated pipeline for identifying phylogenetic marker genes and inferring evolutionary relationships among uncultivated prokaryotic draft genomes

Yu-Wei Wu

Supplementary Information

This Supplementary Information document consists of the following supplementary figures and tables:

- Supplementary Figure S1-S2
- Supplementary Table S1-S7

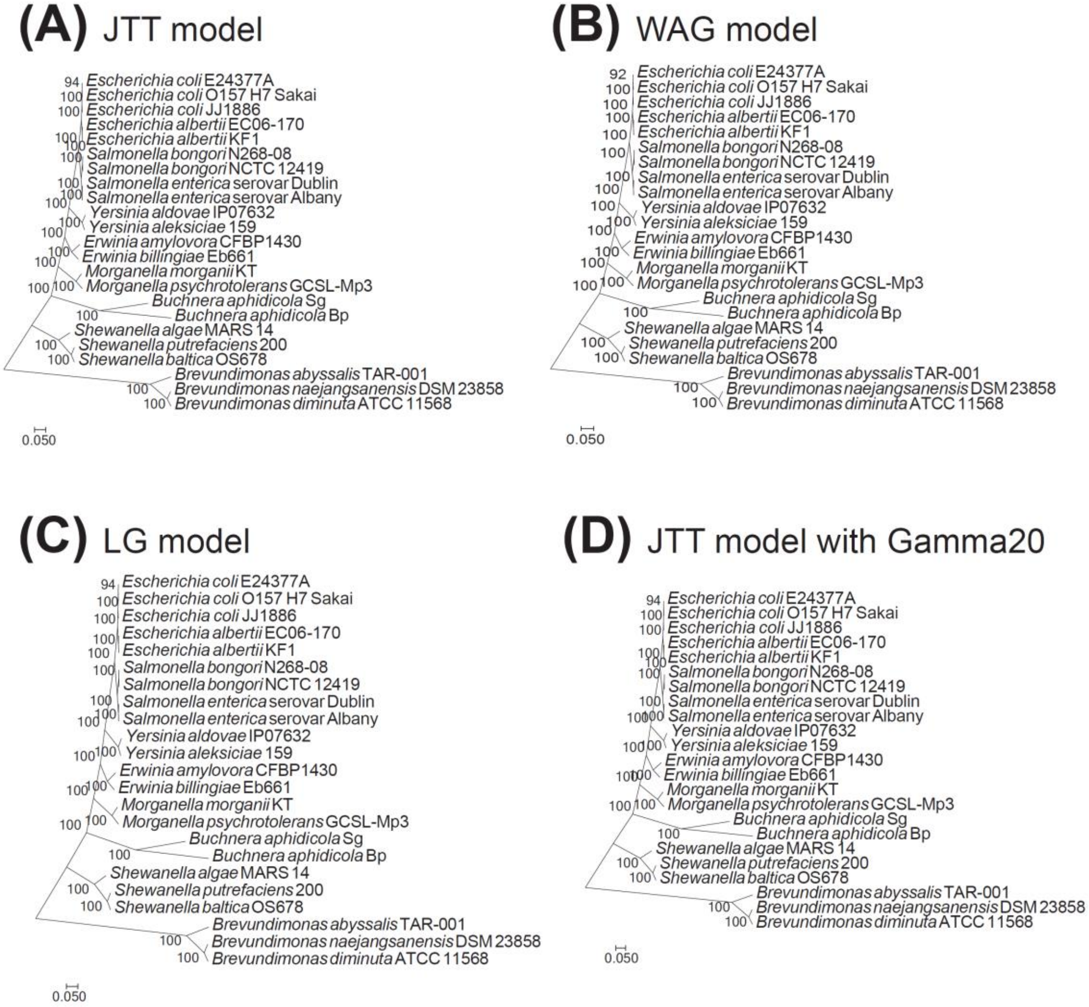

Supplementary Figure S1. The comparison of trees built for the set of Proteobacteria genomes (Supplementary Table S1) using different models provided by FastTree, including (A) JTT model (Jones-Taylor-Thornton 1992), which is the default option of FastTree; (B) WAG model (Whelan and Goldman 2001); (C) LG model (Le and Gascuel 2008); and (D) JTT model with Gamma20 option for rescaling the tree branch lengths.

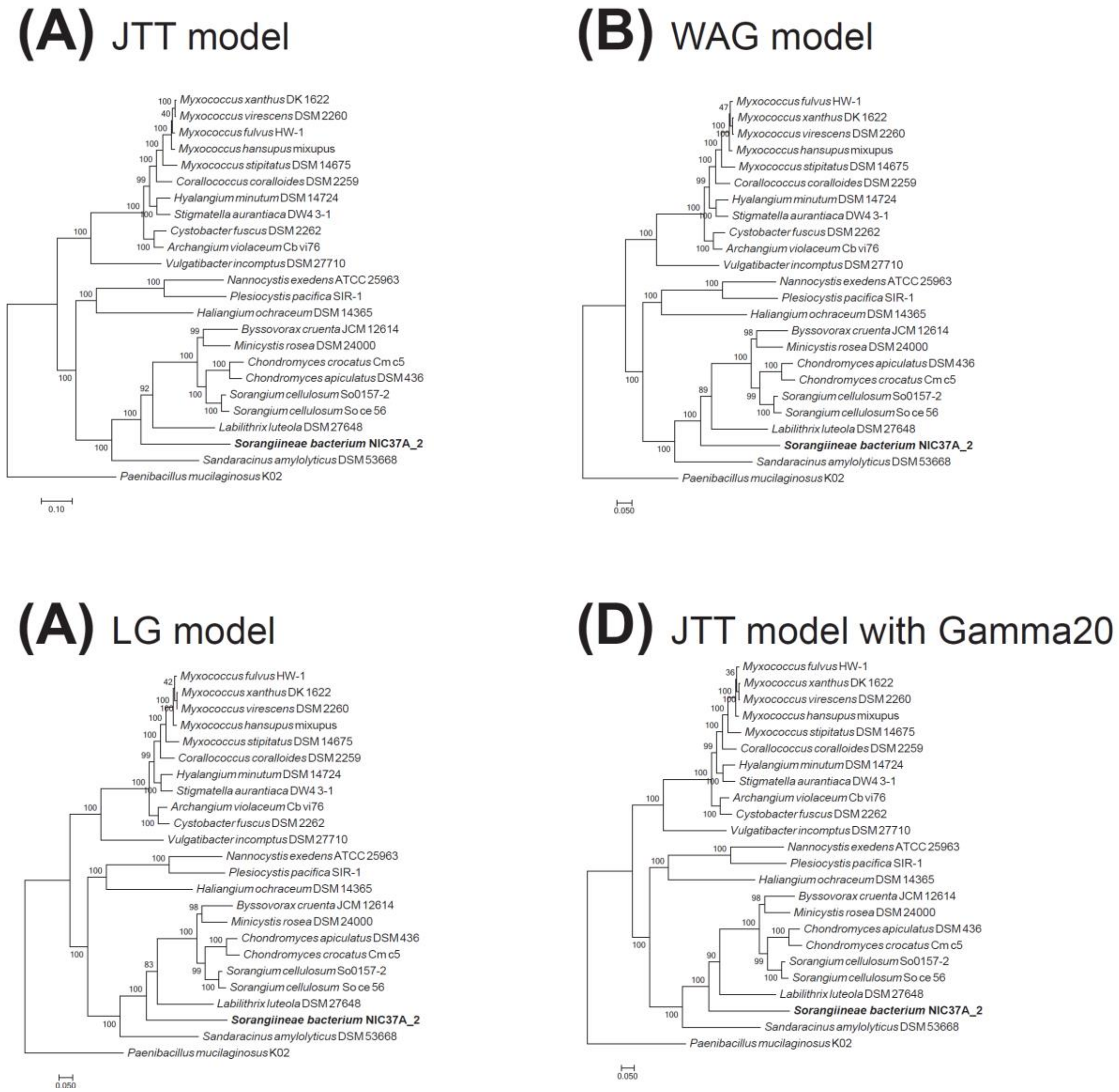

Supplementary Figure S2. The comparison of trees built for the set of Myxococcales genomes (Supplementary Table S6) using different models provided by FastTree, including (A) JTT model (Jones-Taylor-Thornton 1992), which is the default option of FastTree; (B) WAG model (Whelan and Goldman 2001); (C) LG model (Le and Gascuel 2008); and (D) JTT model with Gamma20 option for rescaling the tree branch lengths.

Supplementary Table S1. List of Proteobacteria genomes and their NCBI accession numbers used in the evaluation of ezTree

Species	NCBI accession	Scaffold#
Escherichia coli str. E24377A	NC_009801	1
Escherichia coli str. JJ1886	NC_022648	1
Escherichia coli O157:H7 str. Sakai	NC_002695	1
Escherichia albertii str. EC06-170	NZ_AP014857	1
Escherichia albertii str. KF1	NZ_CP007025	1
Salmonella enterica sp. enterica serovar Albany str. ATCC 51960	NC_CP019177	1
Salmonella enterica sp. enterica serovar Dublin str. CT 02021853	NC_011205	1
Salmonella bongori str. N268-08	NC_021870	1
Salmonella bongori str. NCTC 12419	NC_015761	1
Yersinia aldovae str. IP07632	NZ_CQAX00000000	60
Yersinia aleksiciae str. 159	NC_CP011975	1
Erwinia amylovora str. CFBP1430	NC_013961	1
Erwinia billingiae str. Eb661	NC_014306	1
Morganella morganii sp. morganii KT	NC_020418	1
Morganella psychrotolerans str. GCSL-Mp3 CFSAN046808	LZEX00000000	46
Buchnera aphidicola str. Bp	NC_004545	1
Buchnera aphidicola str. Sg	NC_004061	1
Shewanella algae MARS 14	CDQH00000000	25
Shewanella baltica OS678	NC_016901	1
Shewanella putrefaciens 200	NC_017566	1
Brevundimonas abyssalis TAR-001	NZ_BATC00000000	128
Brevundimonas diminuta ATCC 11568	NZ_ADUI00000000	302
Brevundimonas naejangsanensis DSM 23858	NZ_ATXN00000000	10

Supplementary Table S2. List of Syntrophobacterales genomes and NCBI accession numbers used in inferring the tree for *Smithella* sp. SDB.

Species	NCBI accession	Scaffold#
Desulfacinum infernum DSM 9756	FQVB00000000	81
Desulfobacca acetoxidans DSM 11109	NC_015388	1
Desulfobacca sp. RBG_16_60_12	MGTH00000000	244
Desulfomonile tiedjei DSM 6799	NC_018025	1
Smithella sp. D17	JQOA00000000	271
Smithella sp. F21	JQIE00000000	245
Smithella sp. M82	MAEO00000000	236
Smithella sp. ME-1	AWGX00000000	1037
Smithella sp. SCADC	JQDQ00000000	248
Smithella sp. SC_K08D17	JMED00000000	592
***Smithella* sp. SDB**	LKUC00000000	398
Syntrophaceae bacterium CG2_30_49_12	MNZP00000000	223
Syntrophaceae bacterium CG2_30_58_14	MNZQ00000000	204
Syntrophobacter fumaroxidans MPOB	NC_008554	1
Syntrophus aciditrophicus SB	NC_007759	1
Syntrophus gentianae DSM 8423	FOBS00000000	71
Thermodesulforhabdus norvegica DSM 9990	FOUU00000000	22

Supplementary Table S3. Single-copy marker genes identified for Syntrophobacterales genomes in Supplementary Table S2.

PFAM	Annotation
PF01016.18	Ribosomal_L27, Ribosomal
PF00478.24	IMPDH, IMP
PF00297.21	Ribosomal_L3, Ribosomal
PF03968.13	OstA, OstA-like
PF02677.13	DUF208, Uncharacterized
PF17136.3	ribosomal_L24, Ribosomal
PF00861.21	Ribosomal_L18p, Ribosomal
PF02649.13	GCHY-1, Type
PF00828.18	Ribosomal_L27A, Ribosomal
PF06418.13	CTP_synth_N, CTP
PF05496.11	RuvB_N, Holliday
PF00831.22	Ribosomal_L29, Ribosomal
PF03023.13	MVIN, MviN-like
PF03772.15	Competence, Competence
PF00687.20	Ribosomal_L1, Ribosomal
PF12631.6	MnmE_helical, MnmE
PF08459.10	UvrC_HhH_N, UvrC
PF00410.18	Ribosomal_S8, Ribosomal
PF00673.20	Ribosomal_L5_C, ribosomal
PF01255.18	Prenyltransf, Putative
PF02673.17	BacA, Bacitracin
PF00189.19	Ribosomal_S3_C, Ribosomal
PF00338.21	Ribosomal_S10, Ribosomal
PF00347.22	Ribosomal_L6, Ribosomal
PF01653.17	DNA_ligase_aden, NAD-dependent
PF01176.18	eIF-1a, Translation
PF03946.13	Ribosomal_L11_N, Ribosomal
PF00573.21	Ribosomal_L4, Ribosomal
PF01018.21	GTP1_OBG, GTP1/OBG
PF13393.5	tRNA-synt_His, Histidyl-tRNA
PF00366.19	Ribosomal_S17, Ribosomal

Supplementary Table S4. List of Methanomicrobia genomes and NCBI accession numbers used in inferring the tree for *Methanoculleus* sp. SDB, *Methanolinea* sp. SDB, and *Methanosaeta* sp. SDB.

Species	NCBI accession	Scaffold#
Methanoculleus bourgensis MS2T	NC_018227	1
Methanoculleus chikugoensis isolate L21-II-O	FMID00000000	70
Methanoculleus horonobensis T10	BCNY00000000	15
Methanoculleus marisnigri JR1	NC_009051	1
Methanoculleus sediminis S3Fa	JXOJ00000000	15
***Methanoculleus* sp. SDB**	LKUD00000000	109
Methanoculleus thermophilus CR-1	BCNX00000000	23
Methanolinea tarda NOBI-1	NZ_AGIY02000001	1
***Methanolinea* sp. SDB**	LKUF00000000	509
Candidatus Methanoregula boonei 6A8	NC_009712	1
Methanoregula formicicum SMSP	NC_019943	1
Candidatus Methanosphaerula palustris E1-9c	NC_0011832	1
Methanocalculus sp. 52_23	LGGJ00000000	224
Methanocorpusculum bavaricum DSM 4179	AUMX00000000	33
Methanocorpusculum labreanum Z	NC_008942	1
Methanosaeta concilii GP-6	NC_015416	1
Methanosaeta harundinacea 6Ac	NC_017527	1
Methanosaeta thermophila PT	CP000477	1
***Methanosaeta* sp. SDB**	LKUG00000000	1155
Methanohalobium evestigatum Z-7303	NC_014253	1
Methanosalsum zhilinae DSM 4017	NC_015676	1
Candidatus Methanoperedens nitroreducens ANME-2d	JMIY00000000	10
Candidatus Methanoperedens sp. BLZ1	LKCM00000000	514
Methermicoccus shengliensis DSM 18856	JONQ00000000	17
Methanococcoides burtonii DSM 6242	NC_007955	1
Methanococcoides methylutens MM1	NZ_CP009518	1

Supplementary Table S5. Single-copy marker genes identified for Methanomicrobia genomes in Supplementary Table S4.

PFAM	Annotation
PF04123.12	DUF373, Domain
PF00749.20	tRNA-synt_1c, tRNA
PF00521.19	DNA_topoisoIV, DNA
PF17146.3	PIN_6, PIN
PF07541.11	EIF_2_alpha, Eukaryotic
PF04104.13	DNA_primase_lrg, Eukaryotic
PF04981.12	NMD3, NMD3
PF02002.16	TFIIE_alpha, TFIIE
PF00416.21	Ribosomal_S13, Ribosomal
PF02436.17	PYC_OADA, Conserved
PF01808.17	AICARFT_IMPCHas, AICARFT/IMPCHase
PF01896.18	DNA_primase_S, DNA
PF01864.16	CarS-like, CDP-archaeol
PF04289.11	DUF447, Protein
PF04242.12	DUF424, Protein
PF01920.19	Prefoldin_2, Prefoldin
PF01963.16	TraB, TraB
PF16897.4	MMR_HSR1_Xtn, C-terminal
PF01912.17	eIF-6, eIF-6
PF02996.16	Prefoldin, Prefoldin
PF01242.18	PTPS, 6-pyruvoyl
PF00428.18	Ribosomal_60s, 60s
PF03186.12	CobD_Cbib, CobD/Cbib
PF09886.8	DUF2113, Uncharacterized
PF02654.14	CobS, Cobalamin-5-phosphate
PF00411.18	Ribosomal_S11, Ribosomal
PF00238.18	Ribosomal_L14, Ribosomal
PF01875.16	Memo, Memo-like
PF04476.12	4HFCP_synth, 4-HFC-P
PF09334.10	tRNA-synt_1g, tRNA
PF00410.18	Ribosomal_S8, Ribosomal
PF01092.18	Ribosomal_S6e, Ribosomal
PF00709.20	Adenylsucc_synt, Adenylosuccinate
PF00814.24	Peptidase_M22, Glycoprotease

PF01157.17	Ribosomal_L21e, Ribosomal
PF13685.5	Fe-ADH_2, Iron-containing
PF01725.15	Ham1p_like, Ham1
PF09871.8	DUF2098, Uncharacterized
PF01728.18	FtsJ, FtsJ-like
PF09885.8	DUF2112, Uncharacterized
PF01874.15	CitG, ATP:dephospho-CoA
PF05833.10	FbpA, Fibronectin-binding
PF01090.18	Ribosomal_S19e, Ribosomal
PF01379.19	Porphobil_deam, Porphobilinogen
PF00710.19	Asparaginase, Asparaginase,
PF03367.12	zf-ZPR1, ZPR1
PF01000.25	RNA_pol_A_bac, RNA
PF04609.11	MCR_C, Methyl-coenzyme
PF04475.11	DUF555, Protein
PF01780.18	Ribosomal_L37ae, Ribosomal
PF10369.8	ALS_ss_C, Small
PF00736.18	EF1_GNE, EF-1
PF00701.21	DHDPS, Dihydrodipicolinate
PF01096.17	TFIIS_C, Transcription
PF06026.13	Rib_5-P_isom_A, Ribose
PF02834.15	LigT_PEase, LigT
PF00275.19	EPSP_synthase, EPSP
PF00366.19	Ribosomal_S17, Ribosomal
PF01655.17	Ribosomal_L32e, Ribosomal
PF01994.15	Trm56, tRNA
PF01981.15	PTH2, Peptidyl-tRNA
PF01959.15	DHQS, 3-dehydroquinate
PF01142.17	TruD, tRNA
PF03463.14	eRF1_1, eRF1
PF03911.15	Sec61_beta, Sec61beta
PF00398.19	RrnaAD, Ribosomal
PF00347.22	Ribosomal_L6, Ribosomal
PF13189.5	Cytidylate_kin2, Cytidylate
PF04034.12	Ribo_biogen_C, Ribosome
PF00958.21	GMP_synt_C, GMP
PF06418.13	CTP_synth_N, CTP
PF09920.8	DUF2150, Uncharacterized

PF03737.14	RraA-like, Aldolase/RraA
PF16199.4	Radical_SAM_C, Radical_SAM
PF17214.2	KH_7, KH

Supplementary Table S6. List of Myxococcales genomes and NCBI accession numbers used in inferring the tree for Sorangiineae bacterium NIC37A_2.

Species	NCBI accession	Scaffold#
***Sorangiineae bacterium* NIC37A_2**	NASX00000000	150
Archangium violaceum Cb vi76	JPMI00000000	431
Chondromyces apiculatus DSM 436	ASRX00000000	182
Chondromyces crocatus Cm c5	NZ_CP012159	1
Corallococcus coralloides DSM 2259	NC_017030	1
Cystobacter fuscus DSM 2262	ANAH00000000	76
Haliangium ochraceum DSM 14365	NC_013440	1
Hyalangium minutum DSM 14724	JMCB00000000	44
Labilithrix luteola strain DSM 27648	CP012333	1
Minicystis rosea DSM 24000	CP016211	1
Myxococcus fulvus HW-1	NC_015711	1
Myxococcus hansupus mixupus	NZ_CP012109	1
Myxococcus stipitatus DSM 14675	NC_020126	1
Myxococcus virescens DSM 2260	FNAJ00000000	57
Myxococcus xanthus DK 1622	NC_008095	1
Nannocystis exedens ATCC 25963	FOMX00000000	175
Paenibacillus mucilaginosus K02	NC_017672	1
Pajaroellobacter abortibovis BTF92-0548A 99-0131	NZ_CP016908	1
Plesiocystis pacifica SIR-1	ABCS00000000	237
Sandaracinus amylolyticus DSM 53668	NC_CP011125	1
Sorangium cellulosum So0157-2	NC_021658	1
Sorangium cellulosum So ce 56	NC_010162	1
Stigmatella aurantiaca DW4 3-1	NC_014623	1
Vulgatibacter incomptus DSM 27710	NZ_CP012332	1

Supplementary Table S7. Single-copy marker genes identified for Myxococcales genomes in Supplementary Table S6.

PFAM	Annotation
PF00380.18	Ribosomal_S9, Ribosomal
PF01208.16	URO-D, Uroporphyrinogen
PF02934.14	GatB_N, GatB/GatE
PF01649.17	Ribosomal_S20p, Ribosomal
PF00164.24	Ribosom_S12_S23, Ribosomal
PF06071.12	YchF-GTPase_C, Protein
PF00252.17	Ribosomal_L16, Ribosomal
PF00338.21	Ribosomal_S10, Ribosomal
PF00237.18	Ribosomal_L22, Ribosomal
PF01430.18	HSP33, Hsp33
PF00834.18	Ribul_P_3_epim, Ribulose-phosphate
PF03947.17	Ribosomal_L2_C, Ribosomal
PF02445.15	NadA, Quinolinate
PF00113.21	Enolase_C, Enolase,
PF00572.17	Ribosomal_L13, Ribosomal
PF01148.19	CTP_transf_1, Cytidylyltransferase
PF00809.21	Pterin_bind, Pterin
PF04079.15	SMC_ScpB, Segregation
PF00276.19	Ribosomal_L23, Ribosomal
PF00366.19	Ribosomal_S17, Ribosomal
PF01923.17	Cob_adeno_trans, Cobalamin
PF03946.13	Ribosomal_L11_N, Ribosomal
PF03948.13	Ribosomal_L9_C, Ribosomal
PF00453.17	Ribosomal_L20, Ribosomal
PF03840.13	SecG, Preprotein
PF00189.19	Ribosomal_S3_C, Ribosomal
PF00677.16	Lum_binding, Lumazine
PF03255.13	ACCA, Acetyl
PF00406.21	ADK, Adenylate
PF00416.21	Ribosomal_S13, Ribosomal
PF01379.19	Porphobil_deam, Porphobilinogen
PF02130.16	UPF0054, Uncharacterized
PF13507.5	GATase_5, CobB/CobQ-like
PF00673.20	Ribosomal_L5_C, ribosomal

PF00119.19	ATP-synt_A, ATP
PF05496.11	RuvB_N, Holliday
PF00885.18	DMRL_synthase, 6,7-dimethyl-8-ribityllumazine
PF00687.20	Ribosomal_L1, Ribosomal
PF02576.16	DUF150, RimP
PF00327.19	Ribosomal_L30, Ribosomal
PF02616.13	SMC_ScpA, Segregation
PF01197.17	Ribosomal_L31, Ribosomal
PF08442.9	ATP-grasp_2, ATP-grasp
PF08459.10	UvrC_HhH_N, UvrC
PF00889.18	EF_TS, Elongation
PF00342.18	PGI, Phosphoglucose
PF10150.8	RNase_E_G, Ribonuclease
PF00831.22	Ribosomal_L29, Ribosomal
PF00318.19	Ribosomal_S2, Ribosomal
PF03477.15	ATP-cone, ATP
PF00203.20	Ribosomal_S19, Ribosomal
PF01245.19	Ribosomal_L19, Ribosomal
PF00861.21	Ribosomal_L18p, Ribosomal
PF01250.16	Ribosomal_S6, Ribosomal
PF00466.19	Ribosomal_L10, Ribosomal
PF00562.27	RNA_pol_Rpb2_6, RNA
